# Managing creativity and compliance in the pursuit of patient safety

**DOI:** 10.1186/s12913-019-3935-2

**Published:** 2019-02-12

**Authors:** Sharon H. Kim, Sallie J. Weaver, Ting Yang, Michael A. Rosen

**Affiliations:** 1 Johns Hopkins University Carey School of Business, 100 International Drive, Baltimore, MD 21202 USA; 2 Department of Anesthesiology & Critical Care Medicine, Johns Hopkins University School of Medicine, 750 E. Pratt Street, Baltimore, MD 21202 USA; 3 Department of Medicine, Division of Rheumatology, Lyme Disease Research Center, Johns Hopkins University School of Medicine, Baltimore, USA

**Keywords:** Creativity, Compliance, Patient safety, Organizational climate

## Abstract

**Background:**

Are creativity and compliance mutually exclusive? In clinical settings, this question is increasingly relevant. Hospitals and clinics seek the creative input of their employees to help solve persistent patient safety issues, such as the prevention of bloodstream infections, while simultaneously striving for greater adherence to evidence-based guidelines and protocols. Extant research provides few answers about how creativity works in such contexts.

**Methods:**

Cross-sectional survey data were collected from employees in 24 different U.S.-based outpatient hemodialysis clinics. Linear mixed-effects models were utilized to test study hypotheses. Professional status, clinic climate variables, and interaction terms were modeled as fixed effects, with a random effect for clinic included in all models.

**Results:**

Our results show that high status employees contributed more creative patient safety improvement ideas compared to low status employees. However, when high status employees were part of clinics with a stronger safety climate of compliance, they contributed fewer creative ideas compared to their counterparts working in clinics with a reduced compliance orientation. We also predicted low status employees working in less punitive clinics would contribute more creative ideas, but this hypothesis was not fully supported.

**Conclusions:**

This study suggests that in hospitals and clinics that rely on strict protocols and formal hierarchies to meet their goals, the factors that promote creativity may be distinctively context-dependent. Implications for theory, practice, as well as future directions for research examining creativity in healthcare and safety critical contexts are discussed.

**Electronic supplementary material:**

The online version of this article (10.1186/s12913-019-3935-2) contains supplementary material, which is available to authorized users.

## Background

Creativity, the production of novel and useful ideas [1, 2] is a clear leadership priority in the modern health care organization [3, 4]. Though forward-thinking healthcare leaders may be motivated to facilitate creativity among their clinicians and front-line staff, it remains largely unclear how to most effectively achieve such outcomes. Extant research in management and organizational behavior reveals several important factors that positively influence employee creativity such as perceived leader support [5], empowering leadership style [6] and organizational culture and climate [7]; however, little work to date examines the influence of such factors in clinical settings, that, unlike many business organizations, do not typically follow a mission centered around creativity.

Much of the existing empirical work to date focuses on organizations in sectors focused on strategic innovation, such as technology firms and research and development laboratories. These types of organizations typically try to provide an environment conducive to individual creativity. For example, employees may be provided with greater opportunities for autonomous work [8], a factor shown to encourage workplace creativity [9]. While these types of provisions are possible in some organizations for certain types of roles, great autonomy is not typically characteristic of many of the job roles in safety critical settings, such as health care facilities. Most hospitals and clinics rely on highly specified behavioral protocols to run safely and efficiently (e.g., pre- and post-operative checklists, hand hygiene, catheter insertion, maintenance, and removal bundles protocols). Behavioral protocols have certain advantages in that they can limit the margin of error for certain processes and procedures thereby helping to promote or maintain safety. However, research has shown that limiting the behavior of individuals this way can have negative consequences on the individual’s creative performance [9]. Furthermore, while these procedures do provide important guidelines for practice standardization, many problems they aim to address continue to remain unsolved [10]. The persistence of such issues has prompted many organizations to search for new, creative solutions; however, progress has been slow [11, 12].

In addition to the strict behavioral protocols that often underlie approaches to safety, the hierarchical orientation of health care settings is another factor that may influence creative problem solving ability [13]. Hospitals and clinics have formal hierarchies with salient differences in the relative professional status of roles [14]. These roles are often well defined and dictated by factors such as education, licensure, and job title. In other words, it is uncommon for any single individual or role to have complete knowledge of all care processes that unfold daily in any single care area, clinic, or department. Therefore, when solving patient safety problems, not having the input of individuals of differing status might be a significant hindrance because only certain individuals may be able to provide key details related to local work processes, inefficiencies and/or vulnerabilities.

The pursuit of creative solutions to clinical patient safety problems in healthcare presents an interesting quandary. Creativity in this context could be thought of in terms of Reason’s description of a person versus system approach to human fallibility [15]. According to Reason, a *person approach* focuses squarely on individual errors and the blaming of individuals in an effort to achieve safety; whereas, a *system approach*, accepts human error as a given and focuses on the conditions under which people work as the most effective levers of safety. In terms of creativity, the *system approach* allows for individuals to remain open and respond to what they see and experience. In sharp contrast, the *person approach* extinguishes any incentive to think or behave differently, which is a prerequisite for identifying novel and useful ideas and solutions to problems.

Other factors that are inherent to organizations, such as strong hierarchies, can also produce unintended barriers to creativity. In theory, hierarchical organizations with safety climates that strongly reinforce routinization and procedural consistency may unintentionally inhibit the creative potential of employees necessary to identify and implement solutions to complex problems [16–18]. Research has shown that employee creativity can be difficult to facilitate even in organizations that actively encourage members to innovate [19, 20]. Therefore, the question of how creativity is affected in highly proceduralized, safety-critical environments is an important empirical question explored in this study.

To this end, we hypothesized and tested several factors that may influence the creative participation of clinicians and staff working in outpatient hemodialysis clinics. First, we examined the effect of professional status on creativity. Our prediction was that low status clinical staff would contribute less creative ideas concerning patient safety opportunities compared to high status clinicians. We also explored two dimensions of organizational safety climate hypothesized to influence creativity among higher and lower status staff based upon theoretical relationships proposed in Vogus, Sutcliffe, and Weick’s [21] model of organizational safety and well accepted definitions and measures of creative problem solving from management and organizational behavior research [22]. Specifically, we predicted that high status clinicians working in clinics with stronger supervisory expectations about safety would perform less creatively than those working in clinics with climates that were less compliance oriented, and low status clinical staff would perform more creatively in clinics with a safety climate that is less punitive in response to error compared to clinics with more punitive climates.

## Methods

### Research setting and procedures

Our study was conducted among a sample of outpatient hemodialysis clinics participating in a quality improvement initiative designed to reduce clinic-level bloodstream infection (BSI) rates. Hemodialysis clinics are a novel safety critical setting in which to study creativity because of their participation in a growing national movement to reduce the incidence of blood-stream infections experienced by patients undergoing this treatment [23]. Significant human and financial resources have been put toward overcoming this challenge [24, 25], making it an excellent example of an organizational safety problem in need of creative solutions.

Hemodialysis is a 3 to 5-h blood filtering treatment received three times per week by individuals who suffer from chronic kidney disease or kidney failure. When receiving treatment, individuals are connected to a dialyzer machine via one of three methods: fistula, graft or catheter [26]. Between 60 and 80% of patients initiate dialysis via catheter, a method that is particularly vulnerable to infection and yields higher rates of patient morbidity and mortality [10, 27]. The CDC estimates that 37,000 catheter-related bloodstream infections occur every year at an average cost of $23,000 per hospitalization, and up to a quarter of these infections ultimately result in death [10]. Interventions to reduce the incidence of these infections have been developed and demonstrated success in hospital-based critical care settings [25]; however, outpatient dialysis clinics have not yet realized the same reductions in infection rates. Therefore, many clinics have attempted to utilize quality improvement interventions designed to elicit ideas from their staff to implement in practice.

Cross-sectional survey data were collected from clinical staff working in 24 different hemodialysis clinics participating in this longitudinal quality improvement initiative [24]. All clinics were in major metropolitan areas in the United States. Data from three clinics were excluded from the analyses because they either withdrew from the project or had less than 10% staff participation. Two individual participants were removed from our analyses because they worked with more than one clinic in the sample. All other individuals in the sample worked exclusively at a single clinic. Our final sample included 229 respondents nested in 21 separate clinics.

### Measures

#### Creativity

Our dependent variable, creativity, was assessed using an adapted version of the Staff Safety Assessment (SSA) [28]. The SSA asks clinic staff to individually generate ideas for patient safety and quality improvement. Specifically, participants were asked to identify “the most important patient safety opportunity” in their clinical area. Two expert coders familiar with the operations of hemodialysis clinics coded the entire sample of ideas (*n =* 229) for novelty and usefulness [1, 2] on a 5-point Likert scale (1 = strongly disagree, 5 = strongly agree). Coders were selected based on their expertise in safety and quality improvement efforts in outpatient dialysis settings. Coders were blind to our hypotheses and any potentially identifying respondent information including job title and location. A single creativity score was created for each response by multiplying the novelty score and the usefulness score [29, 30]. The highest creativity scores represented ideas that were both novel and useful. During coding, 21 responses were removed due to missing data. Agreement on ratings of novelty (*α* = .81) and usefulness (*α* = .73) was good, and therefore the two coders’ scores were averaged together to form a composite score.

#### Status

Clinical practice regulations require that staff members with less formal education practice under the supervision of clinicians with more formal education. Because education level accurately mirrors the relative differences in position and influence in the clinical care domain, it was used as a proxy indicator of professional status. Status was operationalized in terms of the amount of education beyond high school required to hold a position. This operationalization is consistent with measures used by other researchers who conduct organizational research in clinical care settings [31].

**Fig. 1:**
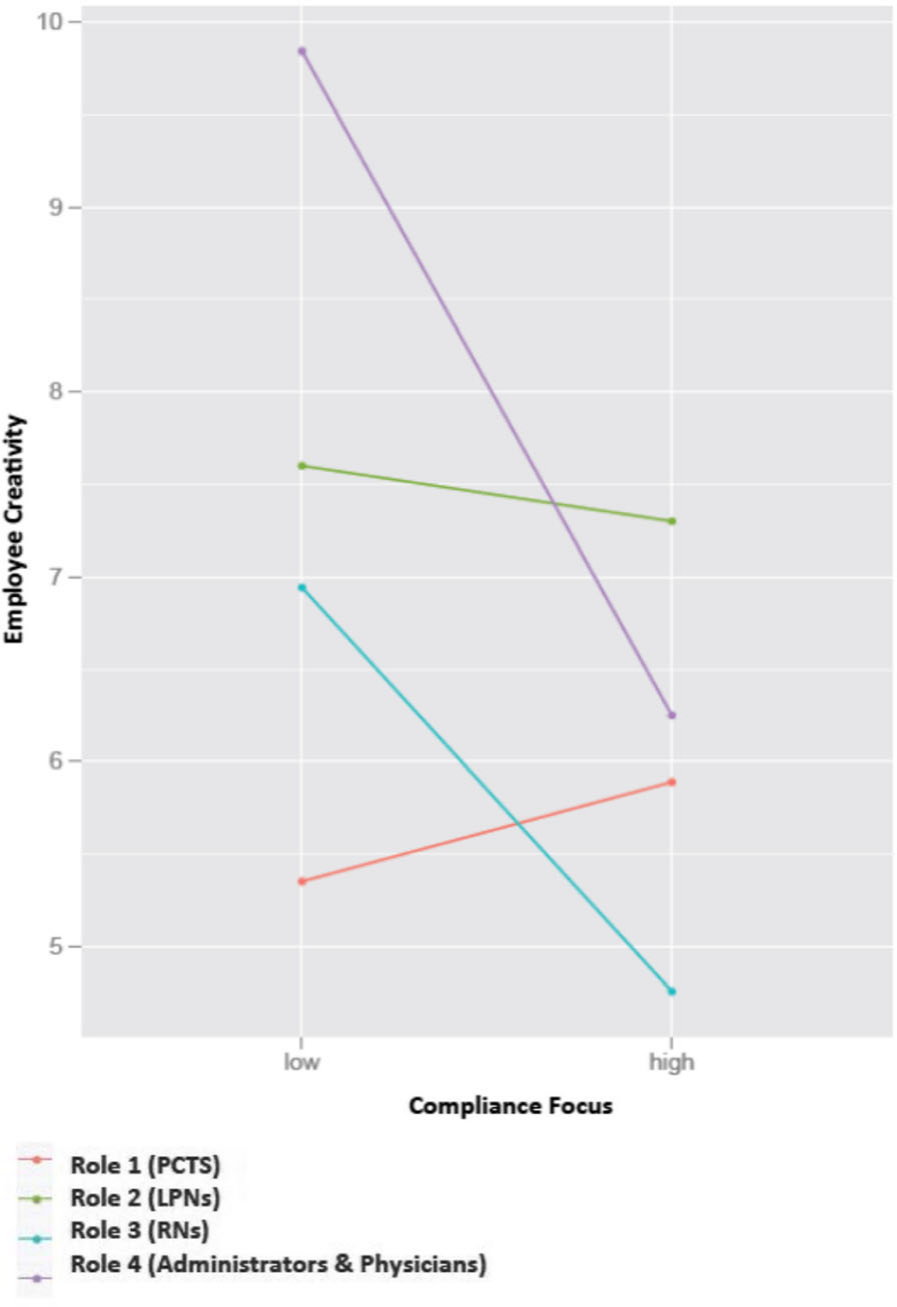
Safety climate was found to moderate the relationship between status and creativity, such that the creativity of ideas contributed by Role 4 clinicians was significantly reduced in clinics with a greater focus on compliance

Participants were categorized into Roles 1, 2, 3, or 4 (See Table 1 and Fig. 1). Role 1 is comprised of patient care technicians (PCTs) who are required to have a high school diploma or equivalent training. Licensed Practical Nurses (LPNs) and Registered Nurses (RNs) were categorized separately in our analyses. LPNs complete less formal education, possess different credentialing, are compensated differently, and have less decision-making ability compared to RNs. Furthermore, RNs tend to mediate between strategy and day-to-day operations whereas LPNs work mostly in the procedural domain [32]. Therefore, in our study, LPNs are categorized as Role 2 and RNs are categorized as Role 3. Clinical staff in Role 4 have education beyond a bachelor’s degree and include physicians and clinic administrators.

**Table 1:** Professional status as operationalized in these analyses

Role	Education	Job Title
1	High School Diploma or Equivalent	Patient Care Technicians
2	Postsecondary Non-Degree Award	Licensed Practical Nurse (LPN)
3	Associates or Bachelor’s Degree	Registered Nurse (RN)
4	Graduate or Post-Graduate Degree	Administrators, Physicians

#### Clinic climate

Clinician and staff perceptions of clinic safety climate included in the dataset were originally collected using a version of the Hospital Survey on Patient Safety Culture (HSOPS) [33] with minimal adaptations for the outpatient hemodialysis setting. The survey asks participants about the culture in their clinic as it pertains to safety, including two dimensions that were hypothesized to moderate the effects of status on individual creativity. Supervisory expectations regarding patient safety and compliance with safety practices was measured using the 4-item composite scale, *Supervisor Expectations and Actions Regarding Safety.* Non-punitive climate was measured by the 3-item composite scale, *Non-Punitive Response to Error*. The specific items comprising these scales and relevant scale properties are reported in the Appendix. Participants used a 5-point Likert-type scale to indicate the degree to which they agreed or disagreed with each survey item (1 = strongly disagree, 5 = strongly agree). The HSOPS has demonstrated sound psychometric properties across a variety of samples and has demonstrated predictive validity for meaningful outcomes, including patient outcomes [34]. The survey was administered to all clinicians and staff electronically using a commercially available online survey platform. The average number of survey respondents per clinic was 11.43 (*SD* = 7.70) and the average survey response rate among the 21 included clinics was 57% (*SD* = 0.32).

In line with survey scoring parameters [33], individual respondent scores were pooled to create a clinic level score on each dimension that reflects the percentage of clinic team members who responded positively on the dimension. Indices of within-clinic agreement supported aggregating individual responses to clinic-level scores for both *Supervisory Expectations* and *Non-Punitive Response to Error.* ICC(1) values ranged from 0.33 to 0.46 and r_WG(j)_ scores [35, 36] ranged from 0.68 to 0.80, indicating reasonable within-clinic agreement (see Appendix).

Additionally, to ensure there was adequate between-clinic variation in clinic-level climate scores to warrant hypothesis testing, we conducted separate Multiple Analyses of Variance (MANOVA) with clinic as the independent variable and the specific items included in each climate dimension entered as dependent variables. Results indicated significant between clinic variation for both climate dimensions (Wilks’ λ_supervisory expectations_ = 0.64, *p* < 0.001 and Wilks’ λ_non-punitive climate_ = 0.76, *p* = 0.005).

#### Infection rate

Clinic infection rates are reported as the number of central venous catheter infections per 100 patient months in line with the CDC’s national surveillance system for hemodialysis-associated infections [37].

#### Analyses

Linear mixed-effects models, also referred to as hierarchical linear models, were utilized to test study hypotheses and account for the nesting of individual respondents within clinics. Professional status, clinic-level climate variables, and interaction terms were modeled as fixed effects, with a random effect for clinic included in all models. In simple terms, mixed-effect linear models enabled regression coefficients to vary from clinic to clinic, and then averages these estimates to obtain a coefficient reflecting the overall effect of the variables of interest on creativity. Analyses were conducted using STATA 12.

## Results

Descriptive statistics and correlations among study variables are displayed in Table 2.

**Table 2:** Descriptive statistics and correlations among study variables

	Mean	S.D.	Median	Min	Max	1	2	3	4
Individual level									
1. Creativity	6.26	3.96				–			
2. Professional status	–	–				0.17*			
Role 1 (*n* = 106)	5.63^a^	3.75	6.00	0	20				
Role 2 (*n* = 20)	7.45	4.66	8.00	0	16				
Role 3 (*n* = 68)	5.88	3.74	6.00	0	16				
Role 4 (*n* = 35)	8.20	3.98	8.00	0	16				
Clinic level									
3. Compliance climate	54.33^b^	14.94						–	
4. Non-punitive climate	82.00	9.22						0.08	–

^a^: Individual-level means reported in this column reflect the raw mean individual-level creativity score by role calculated across all individual respondents in each role. These are reported as unweighted averages (*n* = 189 respondents that provided role data)

^b^: Clinic-level means reported in this column reflect the raw average clinic-level climate score calculated across all clinics (*n* = 21 clinics)

*p < 0.01

Note: Spearman’s rho rank order correlations are reported for associations involving professional status. Pearson’s correlations are reported for all other associations

To test our first hypothesis, a mixed effects model (Model 1) including *Professional Status* as a fixed effect and random effect for clinic was conducted. Results from Model 1 (displayed in the upper portion of Table 3) indicated that status was significantly related to *Clinic Staff Creativity* after accounting for variation between clinics (*χ*^2^ = 13.82, *p* = 0.003). In support of our initial hypothesis, clinical staff in Role 4 exhibited greater creativity compared to staff in Role 1 (*β* = 2.52*,* 95% CI: 1.08, 3.97), however the effect of status was not clearly linear. Post-hoc analyses indicated that Role 1 and Role 2 did not differ significantly (*p* = 0.17), though those in Role 4 exhibited significantly greater creativity than those in Role 3 (*p* = 0.004). This model also suggests that meaningful variance in creativity (13%) was explained at the clinic level. Following these results, supervisory expectations and non-punitive climate were investigated as hypothesized moderators of the observed professional status-creativity relationship.

**Table 3:** Mixed effects models examining the relationship between professional status and creativity, as well as the hypothesized moderation effect of compliance focus

		Creativity			
		Coefficient (SE)	*Z-*Test	95% CI	
Model 1					
Individual level	Constant	5.57 (0.51)	10.84**	4.56, 6.57	
	^t^Role 2	1.60 (0.89)	1.78	-0.15, 3.35	
	^t^Role 3	0.37 (0.59)	0.37	-0.79, 1.53	
	^t^Role 4	2.52 (0.74)	2.52**	1.08, 3.97	
	Random Effects	Variance Component	SE	χ2	*P*
	Constant, *u*_*0*_	1.48	0.39	12.47	0.002
	Level-1, *r*	3.57	0.17		
Model 2					
Individual level	Constant	6.02 (4.86)	1.24	-3.50,15.39	
	^t^Role 2	12. 48 (7.93)	1.57	-3.06, 28.03	
	^t^Role 3	14.88 (6.44)	2.31*	2.27, 27.49	
	^t^Role 4	21.54 (7.61)	2.83**	6.63, 36.45	
Clinic level Interaction	Compliance focus (CC)^tt^	-0.006 (0.06)	-0.11	-0.12, 0.06	
	Role 2* CC	-0.13 (0.09)	-1.38	-0.31, 0.05	
	Role 3* CC	-0.17 (0.08)	-2.27*	-0.32, -0.02	
	Role 4* CC	-0.23 (0.09)	-2.52*	-0.40, -0.05	
	Random Effects	Variance Component	SE	χ2	*P*
	Constant, *u*_*0*_	1.29	0.36	9.33	0.001
	Level-1, *r*	3.49	0.17		

^t^Role 1 status group is the dummy coded reference group

^tt^Climate scores are mean-centered

* *p* < .05, ** *p* ≤ .001

### Omnibus tests of hypothesized moderation effects

To test hypothesized moderation effects of *Supervisory Expectations* and *Non-Punitive Climate* on the observed status-creativity relationship, separate clinic-level simple linear regression models (SLR) were first examined. These models regressed the correlation between status and creativity on each of the climate dimensions. The SLR models provide a preliminary omnibus test of hypothesized moderation effects and were examined prior to conducting more complex mixed effects regression models. This method is commonly used for testing moderation hypotheses in biostatistics. Results suggested that *Supervisory Expectations* (*F* (1,17) = 10.91, *p* = 0.004) significantly moderated the relationship between status and creativity, such that the relationship between status and creativity was significantly reduced as supervisory expectations regarding compliance increased. This provided initial support for moderation Hypothesis 2, and prompted additional exploration regarding the directionality of this effect using mixed effects modeling. The SLR model, however, did not detect a significant moderation effect for *Non-Punitive Climate* (*p* = 0.76); and therefore, we did not find adequate support to suggest further testing of Hypothesis 3.

### Examining the moderating effect of supervisory expectations

We used mixed-effects regression modeling (Model 2) to further explore the moderating effect of supervisory expectations. Results are summarized in the lower \portion of Table 3. In Model 2, creativity was regressed onto status, supervisory expectations, and the interaction between supervisory expectations and status. Clinic was again modeled as a random effect parameter. As seen in the lower portion of Table 3, the interaction model explained significant variance in creativity (*χ*^2^ = 28.52, *p* < .001). In clinics with stronger supervisory expectations regarding compliance, Role 1 staff contributed ideas that were as creative as those ideas contributed by Role 4 staff (*β* = − 0.23; 95% CI: -0.40, − 0.05). More specifically, in these clinics, the creativity of low status clinical staff was slightly higher, but the creativity of high status clinicians was significantly inhibited (see Fig. 1).

#### Supplemental analysis: Supervisory expectations and infection rates

A supplemental analysis was conducted to examine the extent to which clinic-level scores on supervisory expectations related to clinic-level central venous catheter infections rates. A median split was utilized to categorize clinics into low and high supervisory expectations groups. Results of a two-sample *t*-test did not detect any significant differences in patient infection rates between these groups (*p* = 0.59; see Table 4). These findings, in tandem with the moderation analyses, suggest that clinics with higher supervisor expectations regarding safety and compliance did not achieve significantly higher levels of patient safety, though they were creating environments that could potentially reduce the creativity of their higher status team members.

**Table 4:** Two sample t-test comparing clinic-level infection rates between high and low supervisory expectations clinics

	n (clinics)	Mean^a^	SD	t-test	*p*-value
Low supervisory expectations	11	1.36	1.01	−0.22	0.59
High supervisory expectations	10	1.46	1.10		

^a^ Clinic infection rates are report as the number of central venous catheter infections per 100 patient months in line with the CDC’s national surveillance system for hemodialysis-associated infections, p-value is for 1 tailed test

## Discussion

This study examined the influence of professional status and two aspects of safety climate on creativity among clinicians working in outpatient hemodialysis clinics. Our results demonstrate that, as predicted, high status clinicians contributed more creative patient safety improvement ideas compared to low status clinical staff. However, in clinics with stronger supervisory expectations about safety and compliance, the creativity of the two highest status groups (Roles 3 & 4) was dampened. Though we did not specifically predict a drop in the creativity of clinicians in Role 3 (RNs), we find this result interesting and worth further exploration. Some work in management suggests that it is more difficult for middle status members of organizations to be creative due to pressures from above and below [38]. It is possible that middle status conformity pressure interacts with compliance focus in a way that could explain these results more thoroughly. Moreover, the inhibition of creativity was significantly greater for those clinicians of highest status (Role 4). We also predicted that low status staff in clinics operating under a less punitive climate would contribute significantly more creative ideas; however, this hypothesis was not supported.

Our research is not without limitations. First, because our sample was dependent on surveys, the possibility of non-response bias should be noted. It is possible that some individuals did not respond based on potentially meaningful, but unmeasured variables. Additionally, the mix of roles varied across clinics. For example, one clinic did not have any LPNs. Second, the generalizability of our findings may be constrained given our sample of outpatient hemodialysis clinics. It is possible that these results may not be exactly the same in an acute, hospital care setting or another organization operating in a different safety critical industry. It should also be noted that the participating clinics were providing safe care at higher levels compared to national averages [39].^1^ It is possible that the relatively advanced level of care at which these clinics were operating may have influenced the results. For example, in organizations that exhibit poor performance, clinical staff might have many more ideas about how to improve patient safety simply because there is a greater opportunity for improvement. Additional studies with larger or different clinical settings and staff samples could help determine the extent to which these limitations might be of any significant concern.

Furthermore, our findings suggest that there may be diminishing returns on some aspects of organizational performance associated with strong supervisory expectations about safety and compliance. In this sample of clinics, a greater supervisory focus on safety and compliance did not result in a significant difference in infection rates,^2^ but it did reduce creativity among high status clinicians. One potential explanation for these findings is that highly compliance-oriented organizations may inhibit creativity because they shape and reinforce a less mindful approach to safety and to work tasks in general [40–42]. Research by Vogus and Sutcliffe [40] demonstrates that a mindful approach to safety is characterized by proactive and deliberate behavior rather than rote compliance. Employees working in organizations that promote mindfulness in safety tend to think critically about their organization as an entire system, and they are encouraged to think creatively about real or potential areas of risk. This type of approach may permit individuals to express their creativity even under the constraints of highly proceduralized work, whereas an overly compliance-oriented climate may greatly reduce the motivation to be creative [1, 2, 8].

Within health care management and safety research, this work highlights an important opportunity to integrate the topic of creativity as an effective tool in managing the changes that many organizations currently face. Though not necessarily specific to creativity, researchers have also recommended managing the dimensions of the safety critical organizational context in order to meet certain goals. For example, Weick and Sutcliffe [41] encourage deference to legitimate expertise rather than heavy reliance on formal hierarchy when problem solving. Their logic echoes what we found in our results – compliance should be emphasized appropriately but not to the point where it overshadows other important benefits to the organization.

This work also contributes to our understanding of creativity in context by providing additional insight into the effects that certain facet-specific forms of safety climate may have on creativity. In our sample of hemodialysis clinics, a stronger climate of compliance significantly inhibited the creative contributions of high status clinicians. The reduction of creativity in these individuals is noteworthy given that these team members are in positions of influence and increasingly tasked with helping to solve patient safety and quality problems. Moreover, these staff members have a large impact on the local clinical climate. Given these important consequences, we argue that these preliminary results warrant further exploration.

Our prediction about the influence of a non-punitive climate on the creative performance of low status staff yielded null results. This prediction was grounded in existing theory [42] and although our results did not fully support this hypothesis, the results did inspire some pertinent questions for future study. For instance, it is possible that the facilitation and motivation of creativity for low status individuals in this context requires stronger, dedicated psychological interventions. Related research may provide some key clues as to what variables may be important to explore. For example, some research [31] suggests that leaders must both explicitly demonstrate that they value contributions from low status employees and those employees must perceive the environment as being psychologically safe to achieve their stated safety goals. Given their close proximity to patients and responsibilities of direct care tasks, we reiterate our original argument for the investigation of how to facilitate the creative potential of lower status clinical staff in proceduralized organizational contexts. The active participation of these members may help organizations find solutions to pervasive problems concerning preventable patient and consumer harm, and we encourage future studies of this relationship.

### Practical implications and opportunities for future research

Our findings suggest several interesting practical implications as well. First, health care practitioners are seeking creative solutions to challenging problems with little to no context-specific guidance for managing such behavior. For instance, three of the top ten strongly encouraged evidence-based patient safety strategies identified by Shekelle and colleagues [43] included itemized checklists and standardized hand hygiene protocols that, when designed or implemented sans frontline staff input or leadership, could be interpreted as tools focused squarely on compliance and adherence. The opportunity to facilitate the development of creative solutions certainly exists in this domain, but they have not yet made their way into mainstream patient safety practice. Furthermore, what is available by way of traditional organizational research on creativity caters mostly to organizations that are structured in ways that are almost diametrically opposed to the typical safety critical work environment, such as a clinic. Additional research will need to be conducted to identify effective methods and practices for cultivating creativity and implementing innovative solutions to these and other existing problems specific to these types of settings.

Second, future research is necessary to more fully understand and address the barriers to implementing change in clinical care settings that are likely tied to these organizations’ focus on compliance and safety. For example, future studies might examine whether a climate that is highly focused on compliance could influence the cognitive approach that individuals take to identifying, understanding, and investigating potential opportunities for improvement in perhaps unintended ways. Demonstrating that individuals respond differently to certain dimensions of safety climate that are typical of this setting would be an important contribution because it may suggest that information and messaging regarding safety should be tailored specifically to the target audience rather than presented uniformly across an organization as it is often done. It is important for practitioners to be able to gauge the boundary conditions of these effects as it relates to clinical staff and consider actionable ways to achieve high levels of patient safety through simultaneous focus on creative problem solving while maintaining adherence to known, effective solutions.

## Conclusion

This work is presented as an empirical prelude to the continued study of creativity in a variety of safety critical contexts, including health care environments that rely on behavioral and procedural protocols and formal hierarchies to meet their goals. Our results suggest that the roadmap to creativity may look meaningfully different for safety critical organizations like hospitals and clinics. Many of these organizations are actively encouraging creativity, even if it is in the service of another goal (e.g., safety, quality improvement, etc.). Expanding the scope of safety research to encompass this change is necessary to clarify the boundaries of our existing knowledge. By expanding the application of creativity to organizations in these sectors, researchers may be able to have a greater impact to both the field and practitioners.

### Additional file

Additional file 1: STROBE Statement. Checklist of items that should be included in reports of cross-sectional studies. (DOCX 34 kb)

## Appendix

**Table 5:** Survey items and related scale properties

Climate Dimension	# of Items	Items	*α*	ICC(1)	Average r_wg(j)_	Wilks’ Lambda	*F*	*p*
Non-punitive climate	3	1. Teammates feel like their mistakes are held against them.^a^ 2. When an event is reported, it feels like the person is being written up, not the problem.^a^ 3. Teammates worry that mistakes they make are kept in their personnel file.^a^	0.72	0.46	0.68	0.76	1.55	0.005
Supervisor expectations & actions promoting pt. safety	4	1. My supervisor/manager says a good word when he/she sees a job done according to established patient safety procedures. 2. My supervisor/manager seriously considers staff suggestions for improving patient safety. 3. Whenever pressure builds up, my supervisor/manager wants us to work faster, even if it means taking shortcuts.^a^ 4. My supervisor/manager overlooks patient safety problems that happen over and over.^a^	0.66	0.33	0.80	0.64	1.98	< 0.001

* Give information separately for exposed and unexposed groups

Note: An Explanation and Elaboration article discusses each checklist item and gives methodological background and published examples of transparent reporting. The STROBE checklist (Additional file 1) is best used in conjunction with this article (freely available on the Web sites of PLoS Medicine at http://www.plosmedicine.org/, Annals of Internal Medicine at http://www.annals.org/, and Epidemiology at http://www.epidem.com/). Information on the STROBE Initiative is available at www.strobe-statement.org

## Footnotes

1 In terms of organizational safety performance, this sample of clinics was comparable to national indices of patient safety outcomes including BSI (mean catheter-related infection rate in our sample = 3.1 per 100 patient months) versus most recently available national average catheter-related BSI rate for outpatient hemodialysis clinics = 4.2 per 100 patient months [39].

2 We conducted a supplementary analysis to examine the extent to which supervisory expectations was related to clinic-level BSI rates. A median split was utilized to categorize clinics into low and high compliance-focused groups. Results of a two-sample *t*-test did not detect any significant differences in patient infection rates between these groups (*p* = 0.57).
