# Supplementary material for: Managing creativity and compliance in the pursuit of patient safety
Source: BMC Health Serv Res. 2019 Feb 12;19:116. doi: 10.1186/s12913-019-3935-2 (PMC6373136; doi:10.1186/s12913-019-3935-2)
Supplement: Supplementary file 1 — STROBE Statement. Checklist of items that should be included in reports of cross-sectional studies. (DOCX 34 kb) [file 12913_2019_3935_MOESM1_ESM.docx]

STROBE Statement—Checklist of items that should be included in reports of ***cross-sectional studies***

|  | Item No | Recommendation |
| --- | --- | --- |
| **Title and abstract** | 1 | (*a*) Indicate the study’s design with a commonly used term in the title or the abstract **[See Abstract, first sentence of “methods” subheading]** |
|  |  | (*b*) Provide in the abstract an informative and balanced summary of what was done and what was found **[See abstract]** |
| Introduction | | |
| Background/rationale | 2 | Explain the scientific background and rationale for the investigation being reported **[Please see pages 1-2]** |
| Objectives | 3 | State specific objectives, including any prespecified hypotheses **[Please see pages 3-4]** |
| Methods | | |
| Study design | 4 | Present key elements of study design early in the paper **[see Methods, page 3-4]** |
| Setting | 5 | Describe the setting, locations, and relevant dates, including periods of recruitment, exposure, follow-up, and data collection **[Secondary analyses of existing data, described on pages 4-5]** |
| Participants | 6 | (*a*) Give the eligibility criteria, and the sources and methods of selection of participants **[Secondary analyses of existing data, inclusion/exclusion criteria for secondary analyses reported on described on pages 4-6]** |
| Variables | 7 | Clearly define all outcomes, exposures, predictors, potential confounders, and effect modifiers. Give diagnostic criteria, if applicable **[Methods, pages 5-6]** |
| Data sources/ measurement | 8* | For each variable of interest, give sources of data and details of methods of assessment (measurement). Describe comparability of assessment methods if there is more than one group **[Description of Data Set, page 5-6]** |
| Bias | 9 | Describe any efforts to address potential sources of bias **[Interrater Reliability, page 5, 7; Limitations Section, page 10]** |
| Study size | 10 | Explain how the study size was arrived at **[Determined by Participation in QI Initiative, pages 10-11]** |
| Quantitative variables | 11 | Explain how quantitative variables were handled in the analyses. If applicable, describe which groupings were chosen and why **[Methods, pages 5-6]** |
| Statistical methods | 12 | (*a*) Describe all statistical methods, including those used to control for confounding **[Methods/Analyses, pages 3-7]** |
|  |  | (*b*) Describe any methods used to examine subgroups and interactions **[Methods/Analyses, pages 3-7]** |
|  |  | (*c*) Explain how missing data were addressed **[Page 5]** |
|  |  | (*d*) If applicable, describe analytical methods taking account of sampling strategy **[N/A, no weighting used]** |
|  |  | (*e*) Describe any sensitivity analyses **[N/A]** |
| Results | | |
| Participants | 13* | (a) Report numbers of individuals at each stage of study—eg numbers potentially eligible, examined for eligibility, confirmed eligible, included in the study, completing follow-up, and analysed **[N/A due to archival data used; however, survey response rates provided on page 6]** |
|  |  | (b) Give reasons for non-participation at each stage **[Please see page 6]** |
|  |  | (c) Consider use of a flow diagram **[N/A]** |
| Descriptive data | 14* | (a) Give characteristics of study participants (eg demographic, clinical, social) and information on exposures and potential confounders **[Please see Table 1]** |
|  |  | (b) Indicate number of participants with missing data for each variable of interest **[Page 6]** |
| Outcome data | 15* | Report numbers of outcome events or summary measures **[Please see Pages 8-9, Table 2]** |
| Main results | 16 | (*a*) Give unadjusted estimates and, if applicable, confounder-adjusted estimates and their precision (eg, 95% confidence interval). Make clear which confounders were adjusted for and why they were included **[Please see Pages 8-9, Table 2]** |
|  |  | (*b*) Report category boundaries when continuous variables were categorized **[Methods/Analyses, pages 3-7]** |
|  |  | (*c*) If relevant, consider translating estimates of relative risk into absolute risk for a meaningful time period **[N/A]** |
| Other analyses | 17 | Report other analyses done—eg analyses of subgroups and interactions, and sensitivity analyses **[Please see Page 10 (footnote)]** |
| Discussion | | |
| Key results | 18 | Summarise key results with reference to study objectives **[Please see Page 9]** |
| Limitations | 19 | Discuss limitations of the study, taking into account sources of potential bias or imprecision. Discuss both direction and magnitude of any potential bias **[Limitations Section, page 10]** |
| Interpretation | 20 | Give a cautious overall interpretation of results considering objectives, limitations, multiplicity of analyses, results from similar studies, and other relevant evidence **[Please see Pages 13-14]** |
| Generalisability | 21 | Discuss the generalisability (external validity) of the study results **[Please see Pages 10 – 11]** |
| Other information | | |
| Funding | 22 | Give the source of funding and the role of the funders for the present study and, if applicable, for the original study on which the present article is based **[Please see Funding Declaration (prior to manuscript)]** |

*Give information separately for exposed and unexposed groups.

**Note:** An Explanation and Elaboration article discusses each checklist item and gives methodological background and published examples of transparent reporting. The STROBE checklist is best used in conjunction with this article (freely available on the Web sites of PLoS Medicine at http://www.plosmedicine.org/, Annals of Internal Medicine at http://www.annals.org/, and Epidemiology at http://www.epidem.com/). Information on the STROBE Initiative is available at www.strobe-statement.org.
